# Pre-Adolescent Diet Normalization Restores Cognitive Function in Young Mice

**DOI:** 10.3390/jcm12113642

**Published:** 2023-05-24

**Authors:** Wenqian Sun, Hidemasa Okihara, Takuya Ogawa, Hideyuki Ishidori, Eri Misawa, Chiho Kato, Takashi Ono

**Affiliations:** 1 Department of Orthodontic Science, Graduate School of Medical and Dental Sciences, Tokyo Medical and Dental University (TMDU), 1-5-45 Yushima, Bunkyo-ku, Tokyo 1138510, Japan; 2 Department of Oral and Maxillofacial Surgery/Orthodontics, Yokohama City University Medical Center, 4-57 Urafune-cho, Minami-ku, Yokohama 2320024, Japan

**Keywords:** mastication, recovery, cognitive dysfunction, growth and development, hippocampus

## Abstract

Mastication is a fundamental function critical for human health. Controlled by the central nervous system (CNS), it influences CNS development and function. A poor masticatory performance causes cognitive dysfunction in both older adults and children. Improving mastication may prevent cognitive decline. However, no study has determined the period of masticatory dysfunction that impairs children’s later acquisition of cognitive function. Herein, we developed an animal model wherein a soft diet was switched to a normal diet at early and late time points in young mice. We aimed to investigate the impact of restored mastication on learning and memory function. Behavioral studies were conducted to evaluate learning and memory. Micro-CT was used to evaluate orofacial structural differences, while histological and biochemical approaches were employed to assess differences in the hippocampal morphology and function. Correction to a hard-textured diet before adolescence restored mastication and cognitive function through the stimulation of neurogenesis, extracellular signal-regulated kinases, the cyclic adenosine monophosphate-response element-binding protein pathway, and the brain-derived neurotrophic factor, tyrosine receptor B. In contrast, post-adolescent diet normalization failed to rescue full mastication and led to impaired cognitive function, neuronal loss, and decreased hippocampal neurogenesis. These findings revealed a functional linkage between the masticatory and cognitive function in mice during the juvenile to adolescent period, highlighting the need for adequate food texture and early intervention for mastication-related cognitive impairment in children.

## 1. Introduction

Mastication plays an important role in the coordinated development of facial structures [1] and is vital for maintaining life [2]. At present, the diet of the civilized man has become more refined and more processed, requiring little bite force because of reduced masticatory stimulation [3,4], which has significant consequences, especially for jaw growth in children [5].

The regulation of mastication, which involves the coordination of jaw and tongue movements, is initiated by the central nervous system (CNS) [6]. Similarly, input from orofacial structures can stimulate the CNS [7]. The brain–stomatognathic axis was revealed through advancements in brain neuroimaging, which recorded changes in masticatory functional activation and hippocampal structural signatures [8]. Clinical studies of masticatory and cognitive function have shown that a poor masticatory performance is associated with cognitive decline in older people [9,10] and children [11,12]. Some studies have revealed that a reduced masticatory efficiency can contribute to a substantial decline in quality of life [13,14].

Furthermore, animal studies have reported cognitive declines associated with decreased mastication [15,16]. Three primary methods, namely molar extraction, soft diet, and bite raise, have been used to develop laboratory models (mainly of rodents) of reduced mastication [17]. These models are used to assess disturbed masticatory activity by recording jaw movements and muscle activity [18]. Disturbed mastication leads to a reduced performance in behavioral tests that is associated with decreased cellular proliferation in the hippocampus [19].

Emerging evidence has revealed an important role of brain-derived neurotrophic factor (BDNF) and its receptor, tyrosine kinase receptor B (TrkB), in hippocampal memory formation, thereby regulating the connectivity of the neurons in the developing and adult CNS [20,21]. BDNF and TrkB work together to initiate the mitogen-activated protein kinase/extracellular signal-regulated kinase (ERK) signaling pathway, which, in turn, phosphorylates and activates the transcription factor cyclic adenosine monophosphate-response element binding protein (CREB), inducing long-lasting synaptic alterations [22,23]. Several studies have shown that reduced mastication can impair this BDNF/TrkB signaling [2,24].

Based on the literature, improvements in masticatory function may be a new path towards preventing or reversing cognitive decline. Dental prostheses in older patients have successfully reduced the cognitive consequences of masticatory dysfunction [25]. In addition, oral masticatory rehabilitation has helped with memory recovery in aged mice [26,27]. More recently, a study showed that the restoration of food properties (from powder food to normal food) prevented hippocampal pyramidal cell reduction in early adult, senescence-accelerated mice [28]. As the growth period is essential for CNS maturation and general somatic system development, an assessment of these factors during the developmental period would be intriguing. However, only few such studies exist. Moreover, to the best of our knowledge, no systematic review has reported on the association between cognitive function repair and mastication, and the underlying mechanism of this, which can emphasize the biological importance of the critical period for cognitive function restoration by changing the masticatory environment, remains unclear.

This study aimed to investigate the influence of normalized mastication on the cognitive function of mice through switching from a soft diet to a standard diet at pre- and post-adolescent time points. The null hypothesis was that there would be no significant differences, irrespective of the timing. Through this study, we clarified whether the null hypothesis would be rejected or not.

## 2. Materials and Methods

### 2.1. Experimental Animals

**Figure 1:**
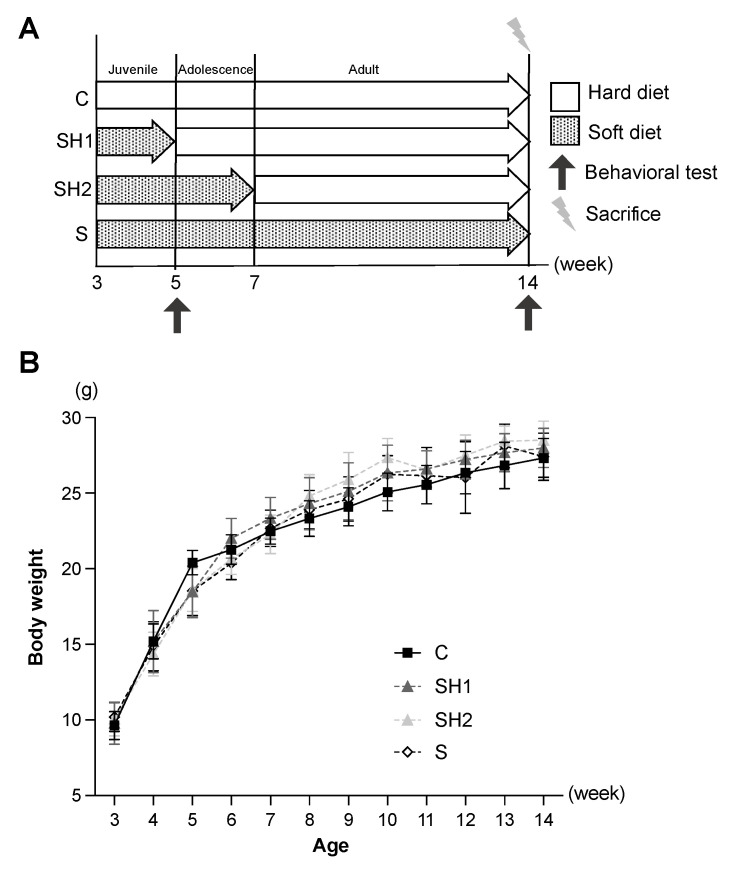
Experimental time points and body weight changes during the study period. (**A**) Timeline of the study period. (**B**) Whole body weight changes from weaning age to sacrifice time in all groups. No significant differences were observed among the four groups (*n* = 25 per group). Error bars denote the mean ± standard error. Abbreviations: C, control group; SH1, Soft–Hard Diet 1 group; SH2, Soft–Hard Diet 2 group; and S, Soft Diet group.

Three-week-old male C57BL/6J mice were purchased from Sankyo Labo Service Corporation (Tokyo, Japan). A priori power analysis was performed to determine the sample size. The mice were randomly categorized into four groups through simple randomization (*n* = 25 mice each, 100 mice in total): a control (C) group, fed with chow pellets (hard diet); the Soft–Hard Diet 1 (SH1) group, fed with powder (soft diet) from 3 to 5 weeks of age and then with chow pellets; the Soft–Hard Diet 2 (SH2) group, fed with powder from 3 to 7 weeks of age and then with chow pellets; and the Soft Diet (S) group, fed only with powder (Figure 1A). The body weight of the mice was measured once a week. All the mice were maintained under specific, pathogen-free conditions. All the procedures complied with the relevant laws and institutional guidelines, and the Institutional Animal Care and Use Committee of Tokyo Medical and Dental University, Japan (approval number A2021-258C2) approved all the animal experiments. All aspects of this study complied with the National Research Council’s Guide for the Care and Use of Laboratory Animals and the ARRIVE (Animal Research: Reporting of In Vivo Experiments) guidelines. No data points were excluded.

### 2.2. Behavioral Tests

The behavioral tests were performed between 5 and 14 weeks of age (Figure 1A).

#### 2.2.1. Passive Avoidance Test

**Figure 2:**
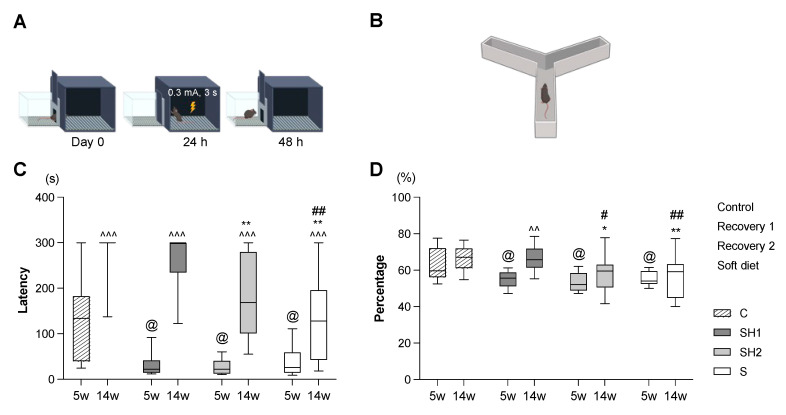
Evaluation of memory and learning function in juvenile and adult mice. (**A**) Passive avoidance test conducted over 3 days. (**B**) Y-maze test in which mice were free to explore for 8 min. (**C**) The latency on the 3rd day in the passive avoidance test. (**D**) The percentage rate in the Y-maze test. Results in (**C**,**D**) show that after 2 weeks of a soft diet, the SH1, SH2, and S groups all showed poor behavioral results. At 14 weeks old, the SH1 group mice showed improved results compared to the S group, while the SH2 group still had poor results. Box edges represent the upper and lower quantiles, with median values indicated by the middle line in each box. Whiskers represent the maxima and minima (*n* = 12 and 16 per group for passive avoidance and Y-maze tests, respectively). ^^^^: *p* < 0.01, ^^^^^: *p* < 0.001 compared with 5-week-old mice in the same group; ^@^: *p* < 0.05 compared with 5-week-old C group mice; *: *p* < 0.05, **: *p* < 0.01 compared with 14-week-old C group mice; ^#^: *p* < 0.05, ^##^: *p* < 0.01 compared with 14-week-old SH1 group mice. Data represented in (**D**) were obtained using a one-way ANOVA followed by Tukey’s multiple comparison test. Abbreviations: C, control group; SH1, Soft–Hard Diet 1 group; SH2, Soft–Hard Diet 2 group; S, Soft Diet group; and ANOVA, analysis of variance. (**A**,**B**) were created with BioRender.com.

The passive avoidance apparatus comprised a light compartment and dark compartment connected by a hole through which the mice could pass [29]. On the first day (0 h), the mice were habituated in the light and dark compartments for 300 s each. On the second day (24 h), the mice were placed in the light compartment and the latency to enter the dark compartment was measured. The mice received an electric shock (0.3 mA) in the dark compartment for 3 s as a conditioning procedure. On the third day (48 h), the latency of the conditioned mice was measured again. The time limit for entry into the dark compartment was 300 s for all three days (Figure 2A).

#### 2.2.2. Y-Maze Test

The Y-maze apparatus comprised three arms (300 × 60 × 150 mm each) joined at 120° angles and randomly designated as “A”, “B”, and “C”. The mice were allowed to explore the three arms freely for 8 min. The number of arm entries was used to determine the percentage of the alternations in the maze based on the ratio of actual-to-possible alternations. Hence, this was calculated using the following equation [29] (Figure 2B):

Percent alternation (%) = [(number of alternations)/(total arm entries − 2)] × 100.

### 2.3. Micro-CT Imaging

**Figure 3:**
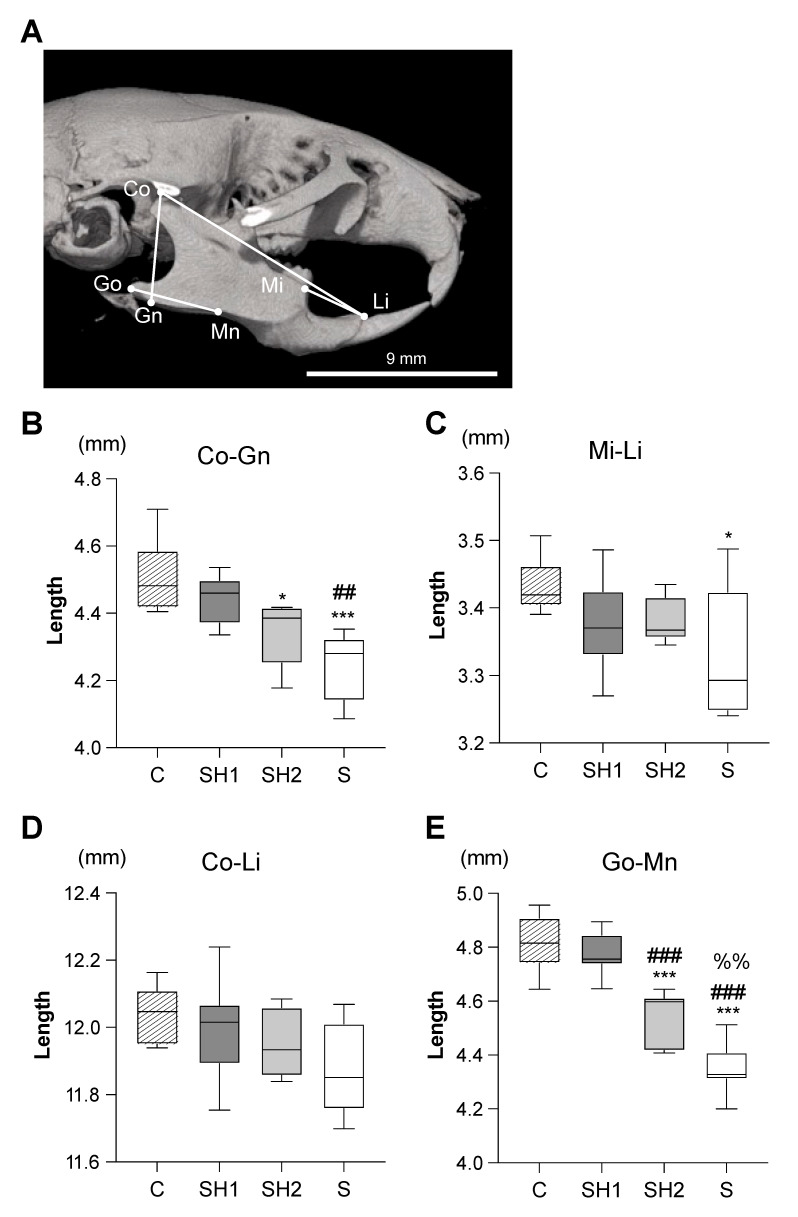
Morphological changes in the mandible induced by altered mastication. (**A**) Landmarks and linear measurements. The ramus height of the mandible (Co-Gn) (**B**) and the anterior (Co-Li) (**C**), whole (Mi-Li) (**D**), and posterior (Co-Mn) (**E**) mandible lengths were measured. The scale bar is shown in the figure. Box edges represent the upper and lower quantiles, with median values indicated by the middle line in each box. Whiskers represent the maxima and minima (*n* = 6 per group). *: *p* < 0.05, ***: *p* < 0.001 compared with 14-week-old C group mice; ^##^: *p* < 0.01, ^###^: *p* < 0.001 compared with 14-week-old SH1 group mice; ^%%^: *p* < 0.01 compared with 14-week-old SH2 group mice. Landmarks: Co, most anterior point of the articular surface of the condyle; Gn, most inferior point of the mandibular angle; Go, most posterior point of the mandibular angle; Mn, ascending ramus dorsal-most ventral point; Mi, most anterior point of the first molar alveolus; and Li, most superior point of the incisor alveolus. Abbreviations: C, control group; SH1, Soft–Hard Diet 1 group; SH2, Soft–Hard Diet 2 group; and S, Soft Diet group.

At 14 weeks of age, the mice were perfused with phosphate-buffered saline (PBS) under isoflurane anesthesia after the behavioral tests. The mouse heads were fixedly immersed in 4% paraformaldehyde (pH 7.4; Wako Pure Chemicals, Osaka, Japan) at 4 °C for 48 h, washed in PBS (pH 7.4) at room temperature and then at 4 °C, and stored in 70% ethanol at 4 °C. A micro-computed tomography (CT) scanner connected to a desktop X-ray micro-CT system (inspeXioSMX-100CT; Shimadzu, Kyoto, Japan), with output settings of 70 kV and 115 μA and a scanning resolution of 8.0 μm, was used to analyze the samples. The landmarks that characterized the cranium morphology were measured using the TRI/3D-BON software (R.9.01.10.4-H, Ratoc System Engineering, Tokyo, Japan) after calibration (Figure 3A).

### 2.4. Nissl Staining

The mouse brains were dissected and categorized into the right and left hemispheres. The left hemisphere was fixed with 4% paraformaldehyde, embedded in paraffin, and cut into 5.0-μm thick coronal sections. The sections were selected from Bregma at −1.46 to −2.30 mm, according to a previous study [30]. The tissue sections were deparaffinized and stained with Nissl stain (cresyl violet acetate, #596-22771; Wako Pure Chemicals, Osaka, Japan). The density of intact neurons per 40,000 μm^2^ in the cornu ammonis (CA)1, CA3, and dentate gyrus (DG) areas of the hippocampus were calculated for a quantitative analysis using ImageJ software (Version 1.53; NIH, Bethesda, MD, USA).

### 2.5. Immunohistochemical Staining

The tissue sections were treated with 3% hydrogen peroxide (Abcam, Cambridge, UK) for 10 min to quench the endogenous peroxidase activity after deparaffinization and rehydration. The sections were incubated with normal goat serum (#06349-64, Nacalai Tesque, Kyoto, Japan) for 10 min at a room temperature of approximately 20 °C to block non-specific binding. Primary antibodies at different specific concentrations were added to the sections, which were incubated overnight at 4 °C with anti-Ki67 antibodies (AB15580, 1:1000 dilution; Abcam). The sections were then washed three times in 1 × Tris-buffered saline with 0.1% Tween 20 (TBST) and incubated with biotinylated secondary antibodies (BA-1000, 1:1000; Vector Laboratories, Newark, CA, USA) for 30 min, followed by processing with an avidin-biotin complex kit (Vector Laboratories) for a further 30 min. The sections were stained with 3,3′-diaminobenzidine (Abcam) and counterstained with hematoxylin. All the stained sections were dehydrated in graded ethanol solutions, cleared in xylene, and mounted using Canada balsam. The number of Ki67+ cells was quantified at 20× magnification using a Nikon ECLIPSE E200 microscope (Nikon Instruments, Melville, NY, USA).

### 2.6. Quantitative Reverse Transcription Polymerase Chain Reaction

The total RNA was isolated from the hippocampus using an RNeasy Plus Universal Mini Kit (QIAGEN, Hilden, Germany), according to the manufacturer’s instructions. The total RNA concentration was updated to 45 ng/μL using a NanoDrop One (Thermo Fisher Scientific, Waltham, MA, USA). First-strand complementary DNA was synthesized from the total RNA through reverse transcription (RT) using the PrimeScript RT Master Mix (Perfect Real Time, #RR036A, Takara, Kusatsu, Japan), according to the manufacturer’s instructions. A quantitative RT polymerase chain reaction (qPCR) was conducted using an Applied Biosystems 7500 Real-Time PCR System (Thermo Fisher Scientific). Amplification was performed for 40 cycles at 95 °C for 3 s and at 60 °C for 30 s using *Bdnf* primers (Mm04230607_s1; Thermo Fisher Scientific), neurotrophic receptor tyrosine kinase 2 (*Ntrk2*) primers (Mm00435422_m1; Thermo Fisher Scientific), and glyceraldehyde 3-phosphate dehydrogenase (*Gapdh*) primers (Mm99999915_g1; Thermo Fisher Scientific). The results were analyzed using the comparative Ct method and normalized to *Gapdh* expression.

### 2.7. Western Blotting

Proteins were extracted from the hippocampus using 20 volumes of RIPA Lysis and Extraction Buffer (#89900, Thermo Fisher Scientific), containing a protease inhibitor and phosphatase inhibitor cocktails (#04080 and #07575-51, Nacalai Tesque). They were loaded onto sodium dodecyl sulfate-polyacrylamide gels for separation. The separated proteins were transferred onto 0.2-μm polyvinylidene fluoride membranes. After blocking the membrane in 5% *w*/*v* bovine serum albumin in 1 × TBST, primary antibodies against phosphorylated (p)-ERK1/2 (1:1000, #9101S; Cell Signaling Technology, Danvers, MA, USA), pan-ERK (1:1000, #610124; BD Biosciences, San Jose, CA, USA), p-CREB (1:1000, #9198; Cell Signaling Technology), CREB (1:1000, #9197S; Cell Signaling Technology), and α-tubulin (1:1000, #2144S; Cell Signaling Technology) were added at 4 °C overnight. After washing in 1 × TBST, the membrane was incubated for 1 h at room temperature with anti-rabbit or anti-mouse secondary antibodies (1:1000, #7074S; Cell Signaling Technology). Signals were detected using enhanced chemiluminescence reagents (#11644, Nacalai Tesque) and the bands were scanned using a ChemiDoc MP Imaging System (Bio-Rad, Hercules, CA, USA) following washing with TBST. A quantitative analysis was performed using ImageJ software (Version 1.53; NIH, Bethesda, MD, USA).

### 2.8. Statistical Analyses

All the data are presented as mean ± standard deviation. Two-group comparisons were performed using a Student’s *t*-test. Multiple comparisons were conducted using one-way analyses of variance (ANOVA), followed by Tukey’s tests. For the non-normally distributed data based on the results of the normality and lognormality tests, equivalent non-parametric tests (the Kruskal–Wallis multiple comparisons test, followed by a Dunn’s multiple comparisons test or paired Mann–Whitney U-test) were performed. GraphPad Prism (version 9.3.0; GraphPad Software, Boston, MA, USA) was used for all the statistical analyses, and a *p*-value < 0.05 was considered as significant.

## 3. Results

### 3.1. Pre-Adolescent Diet Normalization Ameliorated Behavioral Deficits

We conducted the first behavioral tests at 5 weeks of age to examine whether a soft diet could establish and maintain learning, memory loss, or both after an early-stage food texture change. The SH1, SH2, and S groups showed significantly shorter latencies during the passive avoidance test (*p*-value < 0.05 vs. the C group, respectively) (Figure 2C) and lower percentages of alternation in the Y-maze test (*p*-value < 0.05 vs. the C group, respectively) (Figure 2D). In the second round of behavioral tests at 14 weeks of age, the SH1 group showed a noticeable improvement in their learning and memory (*p*-value > 0.05 vs. the C group in both tests). In contrast, the SH2 group (*p*-value < 0.01 vs. the C group in passive avoidance test, and *p*-value < 0.05 vs. the C group in Y-maze test), like the S group (*p*-value < 0.01 vs. the C group, *p*-value < 0.01 vs. the SH1 group in both tests), continued to exhibit impairment. The behavioral differences between the 5- (juvenile) and 14-week-old (adult) mice were analyzed to determine the impact of this diet normalization over time. In the passive avoidance test, the adult mice across all the groups showed a significantly longer latency than the juvenile mice (*p*-value < 0.001 vs. the same juvenile group). In contrast, there were no significant differences in the Y-maze alternation percentages between the adult and juvenile mice, except in the SH1 group, where the percentage improved at 14 weeks (*p*-value < 0.01 vs. the SH1 juvenile group).

### 3.2. Soft Diet-Induced Irreversible Morphological Changes in Craniofacial Structures

No significant differences were noted in the body weights among the groups at any point during the experimental period (Figure 1B). Shape changes associated with the soft diet were observed in the vertical and anteroposterior dimensions of the mandible (Figure 3B–E). The mandible heights (Co-Gn) were significantly decreased in the SH2 (4.343 ± 0.10 mm, *p*-value < 0.05 vs. the C group) and S (4.25 ± 0.10 mm, *p-*value < 0.001 vs. the C group) groups compared to that in the C group (4.51 ± 0.11 mm). The most obvious difference was noted in the posterior lengths of the mandibles (Go-Mn) in the SH2 (4.55 ± 0.10 mm, *p*-value < 0.001 vs. the C group) and S groups (4.35 ± 0.10 mm, *p*-value < 0.001 vs. the C group). The anterior length of the mandible (Mi-Li) was only significantly different between the S (3.33 ± 0.10 mm, *p*-value < 0.05 vs. the C group) and C (3.43 ± 0.04 mm) groups.

### 3.3. Pre-Adolescent Diet Normalization Prevented Neuronal Loss and Dysfunction

**Figure 4:**
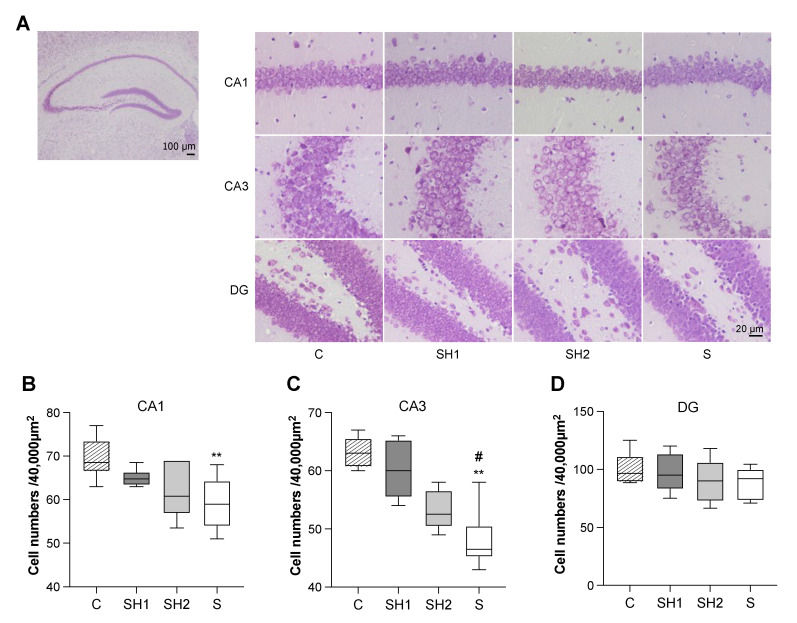
Cell number changes in the hippocampus. (**A**) Nissl staining image of the hippocampus in mice, magnified by 4 × and 40 ×, respectively. Scale bars are shown in the figure. (**B**–**D**) The number of neurons in different mouse hippocampal regions. Box edges represent the upper and lower quantiles, with median values indicated by the middle line in each box. Whiskers represent the maxima and minima (*n* = 6 per group). **: *p* < 0.01 compared with 14-week-old (adult) C group mice; ^#^: *p* < 0.05 compared with 14-week-old (adult) SH1 group mice. All data were assessed with one-way ANOVA followed by Tukey’s multiple comparison test. Abbreviations: C, control group; SH1, Soft–Hard Diet 1 group; SH2, Soft–Hard Diet 2 group; S, Soft Diet group; ANOVA, analysis of variance; CA, Cornu Ammonis; and DG, dentate gyrus.

**Figure 5:**
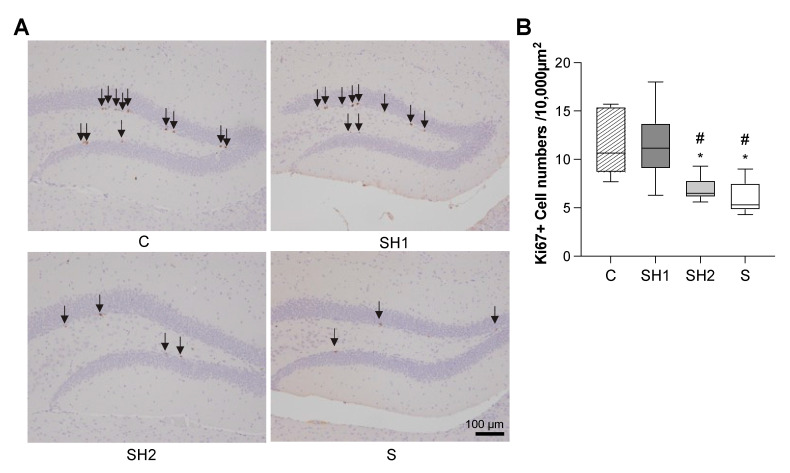
Cellular proliferation activity changes in the hippocampus. (**A**) Representative images of Ki67 immunostaining in the hippocampus of all groups, 10× magnified. Black arrows indicate Ki67+ cells. The scale bar is shown in the figure. Results represented in (**B**) show that the number of Ki67+ cells in the dentate gyrus region of the hippocampus was significantly lower in the SH2 and S groups than in the C and SH1 groups. Box edges represent the upper and lower quantiles, with median values indicated by the middle line in each box. Whiskers represent the maxima and minima (*n* = 6 per group). *: *p* < 0.05 compared with 14-week-old (adult) C group mice; ^#^: *p* < 0.05 compared with 14-week-old (adult) SH1 group mice. Abbreviations: C, control group; SH1, Soft–Hard Diet 1 group; SH2, Soft–Hard Diet 2 group; and S, Soft Diet group.

Nissl staining showed that the neurons were arranged in an orderly way in the hippocampi in the C and SH1 groups. In contrast, the neurons in the SH2 and S groups were scattered in a disorderly way (Figure 4A). Compared to the C group mice, the S group mice exhibited neuronal losses in both the CA1 and CA3 regions (*p*-value < 0.01 vs. the C group, respectively) (Figure 4B,C). Only the CA3 region in the SH1 group showed improvement in its neuron number (*p*-value < 0.05 vs. the S group) (Figure 4C). No significant neuronal differences were identified in the DG regions among the four groups (Figure 4D). Ki67, an endogenous cell-cycle protein commonly employed as a cellular proliferation marker and expressed during all active phases of the cell cycle, was used to examine whether the reductions in the neurons in the CA1 and CA3 regions were because of the impairment of neurogenesis. The number of Ki67+ cells was significantly reduced in both the SH2 (*p*-value < 0.05 vs. the C group and the SH1 group, respectively) and S groups (*p*-value < 0.05 vs. the C group and the SH1 group, respectively), indicating that the long-term soft diet greatly affected cellular proliferation (Figure 5).

### 3.4. Pre-Adolescent Diet Normalization Corrected BDNF/TrkB/ERK/CREB Pathway Impairment

**Figure 6:**
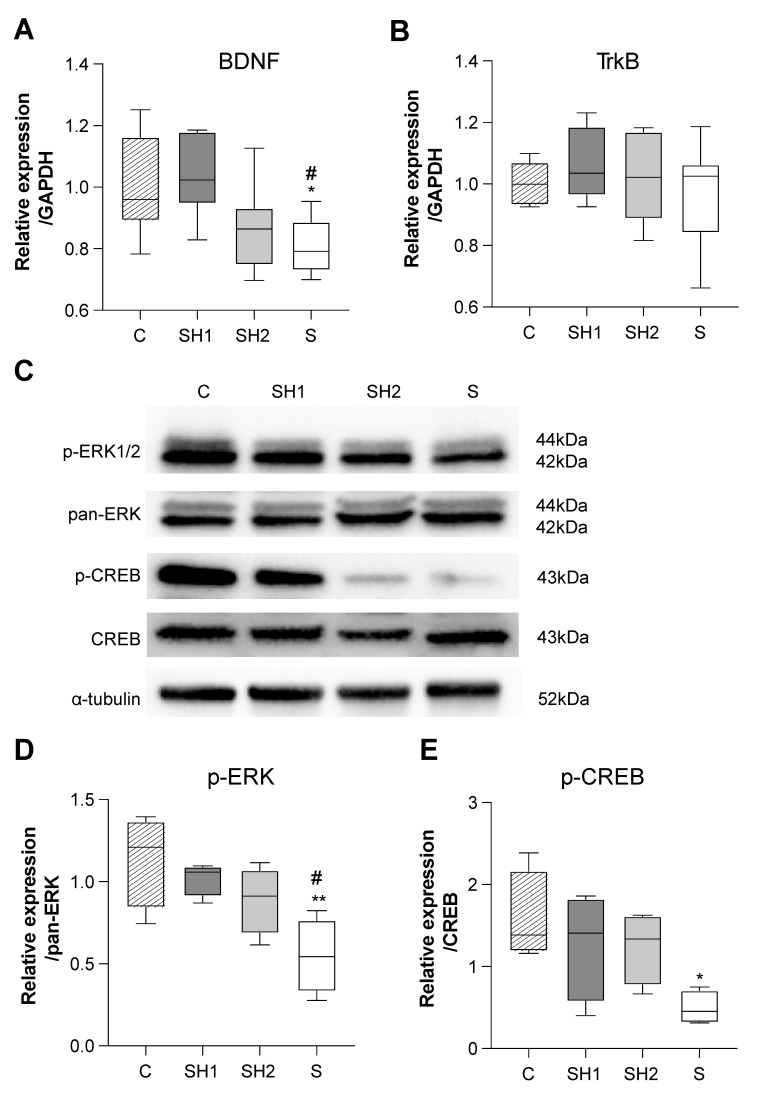
BDNF/TrkB/ERK/CREB signaling changes in the hippocampus. (**A**,**B**) mRNA expression of BDNF and TrkB. GAPDH was used as a loading control (*n* = 8 per group). (**C**) Representative Western blots for p-ERK, pan-ERK, p-CREB, and CREB protein extracts from the hippocampus. α-tubulin was used as a loading control (*n* = 4 per group). (**D**,**E**) Densitometric analysis of p-ERK/pan-ERK and p-CREB/CREB protein levels between groups. GAPDH normalized protein expression. Box edges represent the upper and lower quantiles, with median values indicated by the middle line in each box. Whiskers represent the maxima and minima. *: *p* < 0.05, **: *p* < 0.01 compared with 14-week-old (adult) C group mice; ^#^: *p* < 0.05 compared with 14-week-old (adult) SH1 group mice. Abbreviations: BDNF, brain-derived neurotrophic factor; CREB, cyclic adenosine monophosphate-response element binding protein; GAPDH, glyceraldehyde 3-phosphate dehydrogenase; p-CREB, phosphorylated CREB; p-ERK, phosphorylated extracellular signal-regulated kinase; TrkB, tyrosine kinase receptor B; C, control group; SH1, Soft–Hard Diet 1 group; SH2, Soft–Hard Diet 2 group; and S, Soft Diet group.

In the SH1 group, the BDNF mRNA expression (*p*-value <0.05 vs. the S group) and protein levels of the downstream factors p-ERK1/2 (*p*-value <0.05 vs. the S group) were significantly improved compared to those in the S group, indicating that most of the BDNF/TrkB/ERK/CREB pathway recovered from the short-term reduced masticatory activity associated with the soft diet (Figure 6). However, the SH2 group tended to show decreased BDNF mRNA and p-ERK1/2 and p-CREB protein levels, although these differences were not significant. These findings indicate that reliance on a soft diet progressively infringed upon the activity of the BDNF/TrkB/ERK/CREB pathway. Finally, the BDNF receptor, TrkB (encoded by *Ntrk2*), did not exhibit significant differences among the four groups (Figure 6B).

## 4. Discussion

Mastication plays an important role in the maintenance of hippocampus-dependent cognitive functions. This study investigated the effects of changes in food properties on restoring cognitive function. We found that diet normalization before adolescence could repair cognitive function and memory by preventing impairment in the BDNF/TrkB/ERK/CREB pathway. Conversely, post-adolescent diet normalization failed to repair learning or memory function, resulting in decreased hippocampal neuron numbers and proliferation.

We performed behavioral tests on mice models at 5 weeks of age to test whether a soft diet could induce learning/memory damage from an early stage when the S group had already shown impaired cognitive function. At 14 weeks of age, the behavioral difference between the S and C groups became more apparent. Moreover, the SH1 group showed improved behavior, whereas the SH2 group showed compromised behavior (Figure 2C,D). These results show that impairments in cognitive function are associated with the duration of the soft diet consumption and that the key to the recovery of cognitive function may depend on the timing of the masticatory rehabilitation. Interestingly, when we compared the differences between the two time points in each group, we found that all the mice showed a significantly longer latency time in the passive avoidance test at 14 weeks than at 5 weeks. In addition, significant time interactions in the Y-maze test were only identified in the SH1 group. The passive avoidance test was based on fear-conditioned memory consolidation. The fear memory development peak might appear later than the spatial manipulation peak [31] during the Y-maze. Therefore, early recovery from a soft diet may improve fear memory and repair spatial memory.

The mandible depends more on muscular function to grow, while maxillary growth is independent of this [32]. In this study, the growth of the mandible, as opposed to the growth of the whole craniofacial skeletal structure, was markedly deficient in the mice fed with a soft diet. The most prominent changes were observed in the posterior part of the mandible (Figure 3B–E). These results were expected, as the mice fed with a soft diet acquired a relatively lower functional level of mastication when compared to the mice fed with a normal diet [33], and the direct effects of this soft diet first appeared in the direction in which the masticatory muscle runs [34]. As the food texture hardened before adolescence, which is the critical period of mandible development, the mandibles of the SH1 group mice demonstrated restored masticatory stimulation and had the full potential to grow, as did the C group mice. The SH2 group missed the skeletal developmental peak and optimum learning period for oro-motor behavior [18]. Therefore, there was little potential for the SH2 group to reverse the substantial loss of growth caused by the soft diet. Thus, the mandibles in the SH2 group failed to catch-up in size to the levels seen in the C and SH1 groups.

The hippocampus is the primary region responsible for memory formation. We found remarkable neuronal loss and reduced cellular proliferation (Figure 4 and Figure 5) induced by the reduced mastication caused by the soft diet, thereby corroborating the findings of our previous study [24]. Interestingly, we observed that neurogenesis was repaired in the SH1 group; however, neuron loss was repaired only in the CA3 region. It is possible that, in the SH1 group, restored mastication provided sufficient sensory input to maintain the hippocampal function [35] and repair the neurogenesis and neuronal loss, starting from the CA3 region [36]. The CA3 region is the first to receive signals from the DG region, which serves as a major repository of the neurons for neural stem cells. Moreover, decreased cellular proliferation was observed in the SH2 and S groups. A previous study reported that mastication caused by a long-term soft diet was compensated for by increased chewing cycles [18], indicating that insufficient stimuli reach the hippocampus, leading to decreased cellular proliferation in the DG region.

BDNF/TrkB signaling is crucial for CNS functioning because it regulates neurogenesis, neuronal activity, and synaptic plasticity [37]. In our study, the expression of BDNF mRNA was decreased in the S group (Figure 6A,B), which led to reduced phosphorylation of ERK and CREB (Figure 6D,E), which is in line with the findings of previous studies [38]. Compared to the S group, the SH1 group showed unimpaired/recovered BDNF/TrkB signaling, suggesting that early diet normalization can repair or prevent deficits in the BDNF/TrkB signaling pathway, leading to improvements in behavioral test results. The lower sensory inputs might have affected the sensory afferent development or masticatory central generators in the SH2 and S groups because of a lack of experience with solid diet mastication during the growing period. Degenerative changes could occur in the periodontal ligament mechanoreceptors, where BDNF has also been proven to induce periodontal ligament cell proliferation [36,39]. A recent finding shows that the masseter muscle-derived exosomal neprilysin can be transferred to the hippocampus via the trigeminal nerve during mastication [40]. Therefore, this may help to offer a realistic mechanism between BDNF and mastication in regulating hippocampal memory formation.

This study provides new insights into the relationship between food processing and brain development; a diet mindful of proper masticatory efficiency may be integral for preserving a certain level of hippocampal-based cognitive function. Considering these findings, oral management from an early age (to prevent caries and periodontal diseases) and the orthodontic treatment of malocclusion, both of which restore masticatory function in children, are of considerable importance.

This study has some limitations. First, there is no direct proof that the mastication force was reduced in our study, and morphological and biochemical studies are warranted for the mastication-related muscle. Second, the instant biochemical process by which restored mastication rescues cognitive dysfunction was relatively insufficiently explored. Thus, further studies are expected to explore the bond between mastication and cognitive function.

## 5. Conclusions

Our findings revealed that the restoration of mastication ameliorated cognitive dysfunction by repairing hippocampal neuronal loss and cellular proliferation through the BDNF/TrkB/ERK/CREB signaling pathway. Furthermore, a delay in diet normalization prevents the musculoskeletal system from developing normal functioning; consequently, the deficits in the hippocampus cannot be repaired. These findings may facilitate our understanding of the importance of proper food intake in mastication-related cognitive development.

## Data Availability

The data supporting this study’s findings are available upon reasonable request from the corresponding author.
